# A novel cloning strategy for isolating, genotyping and phenotyping genetic variants of geminiviruses

**DOI:** 10.1186/1743-422X-5-135

**Published:** 2008-10-31

**Authors:** Cica Urbino, Gael Thébaud, Martine Granier, Stéphane Blanc, Michel Peterschmitt

**Affiliations:** 1 CIRAD-UMR BGPI, F-34398 Montpellier, France; 2 INRA-UMR BGPI, F-34398 Montpellier, France

## Abstract

**Background:**

Viruses of the genus *Begomovirus *(*Geminiviridae*) are emerging economically important plant viruses with a circular, single-stranded DNA genome. Previous studies have shown that geminiviruses and RNA viruses exhibit similar mutation frequencies, although geminiviruses are replicated by host DNA polymerases and RNA viruses by their own virus-encoded error-prone RNA-dependent RNA-polymerase. However, the phenotypic effects of naturally occurring mutations have never been extensively investigated in geminiviruses, particularly because, to be infectious, cloned viral genomes usually require sub-cloning as complete or partial tandem repeats into a binary vector from *Agrobacterium tumefaciens*.

**Results:**

Using *Tomato yellow leaf curl virus *(TYLCV), we show here that infectivity can be obtained when only a 41-nucleotide region containing a highly conserved stem-loop is repeated. A binary vector containing this 41-nt region and a unique restriction site was created, allowing direct cloning of infectious monomeric viral genomes provided that they harbour the same restriction site at the corresponding nucleotide position. This experimental system, which can be transferable to other geminiviruses, was validated by analysis of the phenotypic effect of mutations appearing in TYLCV genomes in a single tomato host plant originally inoculated with a unique viral sequence. Fourteen full-length infectious genomes extracted from this plant were directly cloned and sequenced. The mutation frequency was 1.38 × 10^-4 ^mutation per nucleotide sequenced, similar to that found previously for another begomovirus by sequencing PCR-amplified partial sequences. Interestingly, even in this minimal pool of analysed genomes, mutants with altered properties were readily identified, one of them being fitter and reducing plant biomass more drastically than the parental clone.

**Conclusion:**

The cloning strategy presented here is useful for any extensive phenotyping of geminivirus variants and particularly of artificially generated mutants or recombinants.

## Background

Host plant resistance is a major component of integrated pest management (IPM) strategies for the control of virus epidemics; however, its sustainability is often limited by the emergence of resistance-breaking viral strains. The frequency with which such events arise depends among others, on two factors: (i) the error rate of the polymerase with which the viral genome is replicated, and (ii) the percentage of mutations with a positive impact on fitness and/or negative effect on crop production.

The error rate of the RNA-dependent RNA polymerases encoded by RNA viruses has been estimated at 10^-4^–10^-5 ^misincorporation per nucleotide per replication round [1,2]. Small ssDNA viruses, like the parvoviruses of mammals (family *Parvoviridae*) and geminiviruses of plants (family *Geminiviridae*), do not encode their own replicase. Instead, they rely on host DNA polymerases; the rate of errors occurring during replication of these viral genomes is not known. However, the frequency of mutations (number of mutations relative to the consensus, divided by the number of nucleotides sequenced) as well as the substitution rate per year investigated in isolates of geminiviruses [3-5] were found to be of the same order of magnitude as that of RNA viruses. Therefore, it was assumed that the rate of errors generated during the replication of ssDNA genomes was probably higher than the error rate expected for host DNA polymerases. While the distribution of the effect of mutations on viral fitness has been reported for one animal RNA virus [6] and one plant RNA virus [7], thus far no data are available for any ssDNA virus. Furthermore, the distribution and extent of the effect of naturally occurring mutations on virulence has never been investigated in any virus of eukaryotes.

*Tomato yellow leaf curl virus *(TYLCV) is responsible for one of the most damaging begomovirus diseases occurring worldwide [8]. TYLCV was initially described in Israel [9] but is now present in Europe, South East Asia, America and throughout the southern hemisphere. The genome of TYLCV is composed of a small circular ssDNA of 2.79 kb. A mutation frequency of about 3.1–4.1 × 10^-4 ^was recently determined for a distantly related begomovirus from China, *Tomato yellow leaf curl China virus *(TYLCCNV), in viral populations extracted from individual *Nicotiana benthamiana *and tomato (*Solanum lycopersicum*) plants [3]. In this latter study, estimation of the mutation frequency was based on the analysis of numerous PCR-amplified DNA molecules, corresponding to 50% of the viral genome. Although efficient for quantifying mutation frequency, this approach does not allow further investigation of the phenotypic effect of the detected mutation (i.e. fitness and virulence). Indeed, no full-length genomes were analyzed in this particular case, and anyway, the full-length genomes of most geminiviruses are not readily infectious unless using a time consuming construction process.

Although excised monomeric unit length DNA or cloned DNA were found to be infectious for some begomoviruses [10,11] infectivity of most geminivirus genomes can be obtained only if they are delivered to the plant as a full- or partial-length repeat and preferentially by *Agrobacterium tumefaciens *within the T-DNA of a binary vector [12]. Using this technique – commonly known as agroinoculation – it was shown with *Beet curly top virus*, a geminivirus of the genus *Curtovirus*, that recovery of a functional circular viral genome was realised preferentially through replicational release between the repeated origins of replication rather than by homologous recombination [13]. Thereafter, the origin of replication was mapped to a nonanucleotide -TAATATTAC- highly conserved among geminiviruses at the top of a stem-loop structure (SL) of less than 50 nucleotides [14,15]. Taken together these reports suggested that a construction in which the repeated region would be limited to the SL should be infectious. As the nucleotide sequence of the SL is also highly conserved and particularly among begomoviruses, we hypothesised that a binary vector containing only the SL could be used for isolating, genotyping and phenotyping genetic variants of geminiviruses for intra-population studies and experimental evolution.

The present report describes the development of such an experimental system for TYLCV that is easily transferable also to other geminiviruses. The repeated part of the genome generally required for infectivity was trimmed down to a 41-nucleotide fragment containing the SL of TYLCV (33 nucleotides) inserted together with a unique restriction site into a binary vector. An identical restriction site was inserted at the corresponding position in an infectious clone of TYLCV, so that all offspring genomes would be directly clonable in this binary vector and readily infectious upon agro-inoculation with no further manipulation. The value of this system was proved by cloning, sequencing, and characterising the fitness and virulence (here defined as the impact on plant growth) of offspring mutant genomes extracted from an infected plant: we demonstrate the rapid appearance of mutants with enhanced fitness and/or virulence, and moreover we experimentally confirm an elevated mutation frequency for TYLCV.

## Results

### Infectivity of a 1.01 mer TYLCV genome

The binary plasmid containing the 1.01 mer TYLCV genome (p1.01TYLC, see methods) was agroinoculated into tomato plants. Three weeks later, the inoculated plants exhibited typical symptoms of yellow leaf curl and stunting similar to those observed with plants agroinoculated with the tandemly repeated TYLCV-Mld-NotI genome or wild type TYLCV-Mld. Agroinoculation with plasmid pGreen-SL-NotI alone (i.e. with only the 41-nt SL region of TYLCV) produced no symptoms. Thirty-six days after agroinoculation with p1.01TYLC, total DNA was extracted from 10 g of the youngest part of a single symptomatic infected tomato plant. This DNA reacted positively with a TYLCV-Mld-specific probe in Southern blot analysis (data not shown), indicating that the virus had replicated and systemically infected the tomato plant.

This result showed that a very small repeated region of 41 bp, corresponding to the stem- loop structure containing the origin of replication of begomoviruses, is sufficient to allow the release of infectious genomes and further systemic host invasion. As the vector plasmid pGreen-SL-NotI constitutively contains this region, TYLCV genomes with the engineered NotI site could be extracted from the infected plant, and cloned directly back into this vector plasmid in a readily infectious form. To validate this experimental system, we obtained 14 full-length insertions of the viral genome which were fully sequenced. Mutant clones were analysed for fitness and virulence.

### Mutation frequency

**Figure 1:**
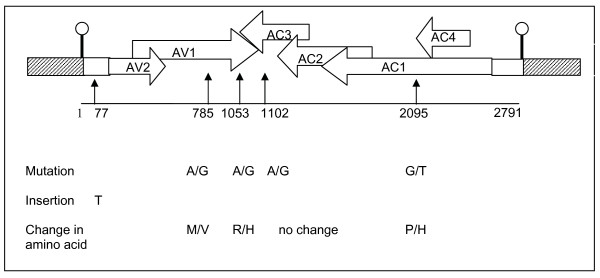
**Schematic representation of the infectious construct p1.01TYLC and position of mutations detected in the progeny**. The schema represents TYLCV-Mild-NotI cloned in the binary plasmid vector pGreen, The hatched boxes represent the vector; white boxes represent the intergenic region of TYLCV-Mld-NotI, and the white horizontal arrows represent viral genes as indicated. The stem loop, including the NotI cloning site, is represented by a vertical bar topped by a white circle. Vertical arrows and associated numbers indicate the position of the mutations detected in the progeny full-length clones. Changes in nucleotide and amino acid sequences detected at the positions indicated are shown below the scheme (original/mutated).

Sequence analysis of the 14 full-length genomes cloned from the tomato plant initially agroinfected with p1.01TYLC revealed that 6 clones differed from the parental sequence either through single substitutions (5 clones) or an insertion (1 clone). A total of 5 mutations were detected throughout the genome (Fig. 1): two non-synonymous mutations in the coat protein gene (AV1), one in the replication associated protein gene (AC1), one synonymous mutation in the replication enhancer protein gene (AC3), and one single insertion in the intergenic region (IR). The AV1 mutation at position 785 was detected in two distinct clones which exhibited 100% nucleotide identity. It is possible that these two clones did not appear independently; therefore this mutant was considered only once in the calculation of the mutation frequency which was thus derived from 13 full-length genomes and was 1.38 × 10^-4 ^mutation per nucleotide sequenced.

### Phenotypic effect of mutations

**Figure 2:**
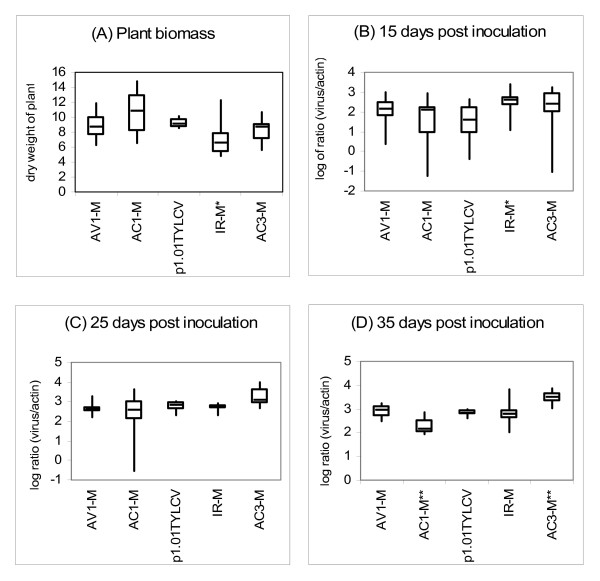
**Biological properties of TYLCV-Mild-NotI and four mutant clones in tomato plants**. Boxplots were obtained from 12 to 14 tomato plants per clone (A) Plant biomass 60 days after inoculation; (B), (C) and (D) Virus quantification normalised to actin2 [log of the ratio of the number of viral copies to that of actin2] 15, 25 and 35 days after inoculation, respectively. Each boxplot shows the median (horizontal line), first and third quartiles (lower and upper limits of boxes), and the minimum and maximum values (delimited by the external whiskers). *: Significant at p = 0.05; **: Significant at p = 0.01.

**Figure 3:**
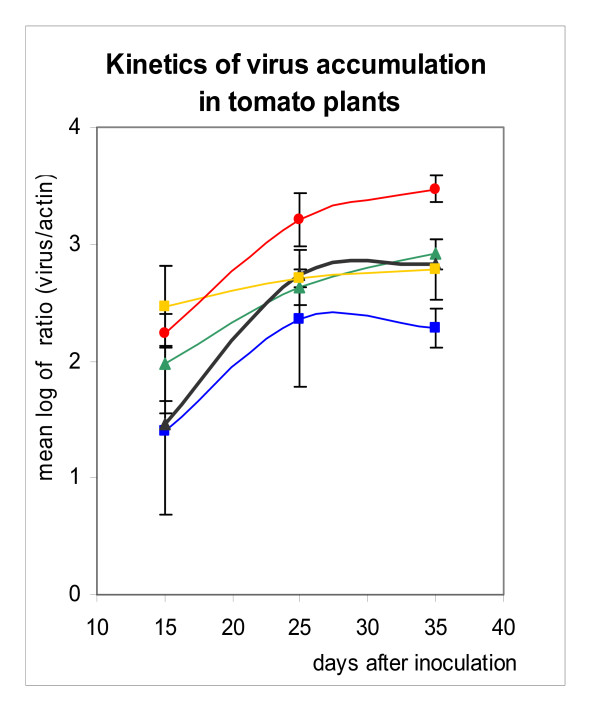
**Average virus accumulation of TYLCV-Mild-NotI and four mutant clones in tomato plants**. Data were obtained from 12 to 14 infected plants per clone. TYLCV-Mild-NotI (black triangle with bold line), AC3-M (red circle), IR-M (yellow square), AC1-M (blue square), IR-M (green triangle). Vertical bars around each point represent the 95% confidence interval.

The IR-, AC1- and AC3-mutants, and one of the AV1-mutants were tested. For each mutant, virus accumulation and its effect on the total dry weight of the infected plants were compared with the parental cloned virus p1.01TYLC. Of 20 tomato plants agroinoculated per clone, 12–14 plants were found to be infected for each clone. All the mutants produced typical yellow leaf curl and stunting symptoms between 17 and 34 days after agroinoculation. Overall, the type of clone had a significant effect on dry weight (p = 7 × 10^-4^), with the mean dry weight of the plants infected with the IR-mutant being significantly lower (p = 0.03) than the mean dry weight of the parental clone (Fig. 2A); no significant deviations from parental clone were recorded with other mutants. The type of clone also had a significant effect on virus accumulation (increasing with time from p = 0.05 to p = 8 × 10^-12^), with the AC3-mutant accumulating more vDNA than the parental clone at 15 and 25 days (p = 0.08 and p = 0.07, respectively; Figs. 2 and 3) and significantly more at 35 days after inoculation (p = 3 × 10^-5^). The IR-mutant accumulated significantly more vDNA at 15 days after inoculation (p = 0.032, Fig. 2B) but was not significantly different at the other time points. The AC1-mutant accumulated significantly less virus at 35 days after inoculation (p = 2 × 10^-4^, Fig. 2D, Fig. 3). No clear correlation could be found between virus accumulation and its effect on the dry weight of the corresponding plant.

## Discussion

Most infectious clones of geminiviruses consist of cloned (partial) tandem repeats of viral genomes, which usually involved tedious, multi-step assemblies of genomic fragments in the construction process. Recently, two groups have reported a simplified cloning strategy of agroinfectious tandem repeats but which only applies to multimeric viral genomes obtained by rolling circle amplification (RCA) using Phi29 DNA polymerase [16,17]. The novel cloning strategy presented here is not only applicable to RCA, but also to PCR amplified genomes or even to replicative forms of the genome directly extracted from infected plants if amplification step is not required or has to be avoided. This will be particularly useful for artificially produced recombinants or mutants derived from already cloned monomeric viral genomes.

**Figure 4:**
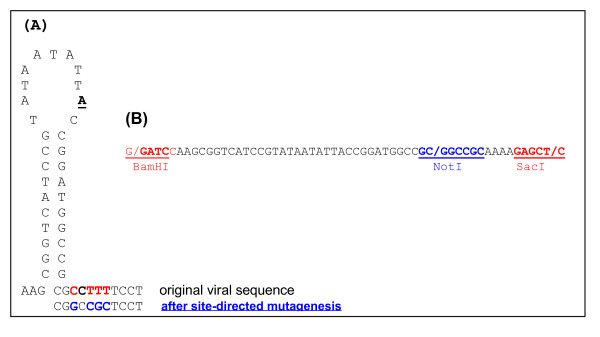
**Construction of pGreen-SL-*Not*I**. (A) Site-directed mutagenesis at the end of the stem loop to generate a NotI site in the TYLCV-Mld clone. The underlined bold A nucleotide in the loop indicates the origin of replication. (B) Oligonucleotide sequence used to insert the stem loop shown in (A) in the multiple cloning site of the vector pGreen.

Based on the traditional technique of partial tandem repeats, we show that infectivity is maintained even when the repeated region was limited to the 41-nucleotide highly conserved stem-loop region of geminiviruses. Moreover, we showed that a binary vector containing this 41-nt region and a unique restriction site can be used to clone infectious monomeric viral genomes provided that they harbour the same restriction site at the corresponding nucleotide position. As previously suggested with longer repeated fragments containing the stem-loop region of two other geminivirus species, the infectivity obtained here is consistent with the mechanism of replicational release [13,14]. Indeed, in our constructs, homologous recombination would only be rarely possible within the tiny 41-nt repeat, which would engender greatly reduced infection success. Thus, it is assumed that the only region of pGreen-SL-NotI vector which is released with the infectious full length genomes is located between the origin of replication and the position of the created cloning site (Fig. 4A). As this released region is identical in about 70% of begomoviruses (comparison with genomic sequences of one member of each of 70 begomovirus species representative of the world diversity,[18]), pGreen-SL-NotI could be used for a large range of begomoviruses provided that NotI is also engineered at the corresponding position of the genomes.

The latent period between inoculation and typical leaf curl observation was similar with both p1.01TYLCV and the tandem construction of TYLCV-Mld-NotI in the binary vector pCambia 2300, but the infection rate was lower with p1.01TYLCV (40% versus 80–90%, data not shown). It cannot be excluded that the lower infection rate was due to the limited repeated region. However with an identical construction in which the cloning site NotI was replaced by XhoI, and the pGreen plasmid was replaced by the binary vector pCambia380, we obtained 90% efficiency of agroinoculation (not shown) suggesting that the reduced length of the repeated region may not necessarily be the reason for the lower infection rate observed with p1.01TYLCV. The restriction sites, either NotI or XhoI, were engineered in a variable region of begomovirus genomes which tolerates mutations without loss of infectivity. The use of an alternative restriction site (here XhoI) may be useful for viral species where a NotI site already exists in the wild type genome. Besides the advantage of directly cloning infectious full-length genomes of a geminivirus, the fact that the cloning site is only 15 nucleotides downstream of the origin of replication contributes to the exclusive cloning of replicable DNAs.

The usefulness of this novel cloning strategy was further demonstrated by showing that not only are TYLCV mutants generated at high rate within 36 days in a tomato plant (5 mutants in 13 clones), but also that they exhibit altered biological properties: some of these mutants accumulating more viral DNA and reducing plant growth more drastically than the progenitor clone. Although this does not represent an exhaustive evaluation of the life traits of the virus, our results suggest that TYLCV mutants with both increased fitness and virulence can appear at a very fast pace, even during a single host infection cycle.

The mutation frequency estimated 36 days after plant inoculation on 13 directly cloned full-length TYLCV DNA genomes (thus precluding any possible PCR amplification artefacts), was 1.38 × 10^-4^. This frequency is in the same order of magnitude as that determined recently [3] with another tomato begomovirus (TYLCCNV). In this earlier study, the authors reported a mutation frequency of 3.1–4.1 × 10^-4 ^at 60 days after infection and of 5.3 × 10^-4 ^at 120 days after infection. The experiment reported here on TYLCV was terminated 36 days after inoculation, i.e. a shorter infection period, which could explain the slightly but significantly lower mutation frequency (p = 0.032, t-test) compared to the 60-day results of TYLCCNV, possibly due to the reduced number of generations. The higher mutation frequency of TYLCCNV [3] may also be explained by (i) the fact that sequencing was not performed on full-length genomes, but on a selected region containing highly variable sequence stretches such as IR and AC1/AC4; (ii) an intrinsic property of the virus; and (iii) to some extent, to the fact that viral sequences were not directly extracted and cloned from infected plants, but first amplified by PCR, which introduces additional mutations at a frequency estimated at around 4.9 × 10^-5 ^[3].

Nevertheless, the results presented here for TYLCV further support a growing body of evidence [2-5,19] paradoxically suggesting a similar mutation frequency in ssDNA viruses and RNA viruses, the former being replicated by supposedly faithful host DNA-polymerases, and the latter by error-prone RNA-dependent RNA-polymerases. This paradox remains unexplained but it has been proposed that the mismatch repair machinery, and/or proof reading activity of DNA-polymerases may not function with the geminivirus genome as it does with cellular DNA [20,21]. Confirmation of this hypothesis lacks important information from both the plant and the virus side. On the one hand, the DNA-polymerase used by ssDNA viruses for replication has not been identified, thus precluding any estimate of its intrinsic error rate. On the other hand, the generation time of ssDNA viruses is not known, again ruling out a formal estimate of the error rate (per nucleotide, per replication round) of the plant polymerase hijacked for virus replication.

After only 36 days of infection, 3 mutant clones exhibiting a phenotype distinct from the parental clone were obtained. To our knowledge, none of the modified positions of the genome have been specifically described in the literature to be involved in molecular interaction at the DNA or protein level. Therefore we can only speculate that the known functions or interactions of the genes and IR promoters in which the insertion and mutations occurred were somehow modified. For example, the higher fitness induced by the single insertion in the IR could be explained by its modified interaction with the transcription activator protein encoded by AC2 [22]. The synonymous mutation in the AC3 gene, which induced significantly higher fitness, could be explained by a higher stability of the mRNA of AC3, which encodes a replication enhancer protein [23]. The AC1 non-synonymous mutation is located in the region of the gene encoding the oligomerisation domain of the Rep protein, which is essential for viral replication, perhaps suggesting modification of the oligomeric state of this protein [24]. Although no evident correlation was detected between fitness (virus accumulation) and virulence (as estimated by the effect on the dry weight of the host plant), the data obtained with the IR mutant indicate that increased accumulation of the virus at the early stage of infection may be damaging for the plant, even if final accumulation levels are unchanged.

## Conclusion

The novel cloning system presented here will be useful for any study in which extensive phenotyping of geminiviruses is required. As illustrated in this paper, it can be used for experimental evolution *in planta*. The technological bottleneck is no longer the time-consuming construction of infectious (partial) tandem clones, but rather the extraction of sufficient amounts of vDNA, particularly for those geminiviruses, such as TYLCV, which accumulate at low concentration in plants. More interestingly the novel cloning strategy will be useful to allow the high throughput phenotyping of artificially generated mutants, as earlier performed in other viral genera [6,7], or even of artificially generated recombinants.

## Methods

### Viral clone and vector plasmid to create agroinfectious unit length viral genomes

A full-length genome of a Réunion isolate of the Mild strain of *Tomato yellow leaf curl virus *(TYLCV-Mld, accession no. AJ865337[25]) previously cloned into a pGEM-T Easy vector, was used in this study. A unique NotI restriction site was created by site-directed mutagenesis (QuikChange^® ^II XL Site-Directed Mutagenesis Kit, Stratagene) at the 3'-end of the conserved stem-loop sequence of the TYLCV-Mld genome (Fig. 4A), generating a clone named pTYLCV-Mld-NotI. The full-length viral genome was released from this plasmid vector by *Bam*HI restriction [25], and subsequently religated to generate circular closed single copies of the TYLCV-Mld-NotI genome.

The binary high-copy plasmid vector pGreen [26] was modified as follows: a 41-bp dsDNA fragment, encompassing the conserved stem-loop (SL) of begomoviruses, and including a NotI site at the same nucleotide position as that described above in TYLCV-Mld, was prepared by hybridisation of two complementary oligonucleotides (Fig. 4B). These oligonucleotides were designed to generate cohesive ends for cloning into the *Bam*HI and *Sac*I restriction sites of the pGreen vector, thus generating pGreen-SL-NotI. Single copies of the TYLCV-Mld-NotI genome (see above) were linearised at the engineered NotI site and cloned into pGreen-SL-NotI, resulting in a 1.01-mer genome. After checking for the sense orientation of the inserted genome, a recombinant plasmid was selected and is referred to as p1.01TYLC (Fig. 1). This plasmid was finally purified from *E. coli *DH5-α, used for all cloning steps, and introduced into *A. tumefaciens *C58 MP90 by electroporation. Transformed *A. tumefaciens *colonies were plated and cultivated at 28°C on LB medium containing kanamycin and gentamycin, and used for agroinoculation as described below.

### Plant inoculation

Tomato plants cv. Nainemor were inoculated at the two-leaf stage by pricking the stem three times at different levels with the tip of an 18 Gauge ×1 1/2 needle previously dipped into a 24 h-plated culture of *A. tumefaciens *carrying p1.01TYLC or derivatives. Plants were maintained in an insect-free containment chamber at 26°C with a 16 h photoperiod. As a negative control, plants were inoculated with *A. tumefaciens *carrying the empty pGreen-SL-NotI plasmid.

### Extraction of viral DNA

Ten grams of leaf and stem material were collected from the youngest part of a tomato plant 36 days after agroinoculation with p1.01TYLC. Total DNA was extracted with the Midi DNeasy plant DNA extraction kit (Qiagen). DNA was eluted in 20 ml of water, and concentrated down to a volume of 500 μl by drying under vacuum for 3–4 h. To eliminate high molecular weight plant DNA, the 1–6 kb DNA fraction containing all the viral DNA forms (vDNA) according to Southern blot assay, was purified from a 1% agarose gel (SV Wizard Gel kit, Promega) and eluted in 80 μl of water. The gel-purified DNA was digested with NotI, microdialysed to eliminate buffer salts, and subsequently used for cloning. The presence of NotI-linearised full-length viral genomes was verified by Southern blotting and probing with a digoxygenin-labelled full-length TYLCV-Mld genome using a DIG-High Prime DNA Labelling and Detection Starter kit (Roche Diagnostics). Positive signals on membranes were revealed with the chemiluminescent substrate CDP star (Roche Diagnostics).

### Cloning of viral progeny

A 7.5 μl NotI-digested vDNA sample was mixed with 300 pg of dephosphorylated NotI digested pGreen-SL-NotI plasmid for a 24 h ligation reaction at 4°C in a final volume of 10 μl. An aliquot of 4 μl of this ligation mixture was then electroporated into 35 μl suspensions of electrocompetent XL1 Blue bacteria (Stratagene) according to the supplier's protocol.

Bacterial colonies were prepared for colony hybridisation with a full-length digoxygenin-labelled TYLCV-Mld genome as described in Sambrook *et al*. [27] and positive signals were revealed as described above. Recombinant plasmids obtained from positive hybridising colonies were digested with NotI to detect the presence and size of inserts on agarose gels, and with *Bam*HI to determine the orientation of the insertion.

### Sequence determination and analysis

Samples of cloned vDNA were sequenced (Genome Express) with four TYLCV-specific primers and two pGreen-specific primers (Table 1). Sequence data were assembled and analyzed with DNAMAN software (version 5.0, Lynnon BioSoft). The mutation frequency was calculated as the number of mutations relative to the parental clone, divided by the number of nucleotides sequenced.

**Table 1:** Primers for detection of TYLCV in infected plants, sequencing of TYLCV clones, and relative quantification of TYLCV with quantitative PCR (Q-PCR)

Primer code	Primer sequence	Final concentration used
**Detection of TYLCV with PCR (600 bp amplicon)**		
		
Ty 2353+	CTGAATGTTTGCATGGAAATGTGC	200 nM
Ty321-	GGTCGCTTCGACATARTCACG	200 nM
		
**Sequencing of TYLCV cloned sequences**		
		
pGreen1589 (+)	CACGACGTTGTAAAACGACG	
pG1825(-)	CACAGGAAACAGCTATGACC	
579 (+)	GATGTTACTCGTGGATCTGG	
1141 (+)	CATGATCAACTGCTCTGATTAC	
1741(+)	GGGCTTCCCGTACTTTGTG	
2321(+)	TGGATTTAGCTCCCTGAATG	
		
**TYLCV primers for Q-PCR (176 bp amplicon)**		
		
Ty 2164+	CTAAGAGCCTCTGACTTACTGC	200 nM
Ty 2339-	AACATTCAGGGAGCTAAATCCAG	200 nM
		
**Actin2 gene primers for Q-PCR (148 bp amplicon)**		
		
Act 1+	CCCRGAGGTHCTCTTCCARC	200 nM
Act 148-	TMCGRTCAGCAATACCAGGG	200 nM

### Phenotypic effect of mutations

Four TYLCV mutant clones, the parental clone p1.01TYLC and the vector pGreen-SL-NotI were each agroinoculated to 20 tomato plants. Prior to agroinoculation, mutant clones that were found to be inserted in the negative sense orientation were released with NotI and re-inserted in the opposite orientation in pGreen-SL-NotI. Plants were maintained in an insect-free containment chamber at 26°C with a 16 h photoperiod. They were randomly distributed in 5 blocks containing each 4 inoculated plants per treatment. Symptoms were monitored visually twice a week from 14 to 60 days after agroinoculation.

The youngest part of the tomato plant has previously been shown to accumulate the highest titres of TYLCV DNA [28], with no significant difference between the four upper leaves [29]. Thus, virus accumulation was monitored using quantitative PCR (Q-PCR) from a foliar disc of 1 cm diameter, collected from the top leaflet of the youngest expanded leaf of each plant at 15, 25 and 35 days after agroinoculation. Total DNA was extracted with the CTAB method [30]. DNA was dissolved in 50 μl of ultrapure water and stored at -20°C until use. Sixty days after inoculation, a leaf sample was collected from each asymptomatic plants to test for the presence of virus by non-quantitative PCR using primers Ty 2353+ and Ty321- (Table 1). Thereafter, on the same day, all the plants were cut at the level of the marks left by cotyledon leaves, dried for 48 h at 60°C, then weighed. Although the reduction of plant biomass was not shown to be a determinant of virulence for the TYLCV/tomato system, it was chosen in this study to estimate the impact of the virus on the fitness of the plant. Indeed, tomato is an annual cultivated plant for which the plant growth has an impact on the yield and is consequently more accurate than the number of viable seeds to estimate the virulence of a viral clone.

### Quantitative PCR conditions

Five microlitres of 1/100 diluted vDNA samples, extracted as described above, were tested for each sample in a final volume of 25 μl, using 96-well optical plates and the Mx3005P^® ^QPCR System (Stratagene). The reaction mix was prepared with the qPCR core kit for SYBR Green I no ROX (Eurogentec) according to the manufacturer's recommendations. The primers designed for TYLCV DNA quantification were Ty 2164+ and Ty 2339-. The tomato actin2 gene was quantified in each extract to allow comparison of vDNA accumulation between DNA extracts based on the same number of plant cells per plant sample. Primers targeting the actin2 gene (Act 1+/Act 148-) were derived from the alignment of five actin2 sequences determined from tomato plants of the cv. Nainemor and sequences available in the GenBank database (Accession nos. U60481, BT013524, BT013707). The sequences and final concentrations of primers are listed in Table 1.

Cycling parameters were 95°C for 10 min followed by 40 cycles of 15s at 95°C, 1 min at 66°C and 30s at 72°C. The amount of specific product was monitored using the dye SYBR Green I. Two replicates were amplified per sample. Data obtained by Q-PCR were analyzed using MxProv4.

### Accumulation of TYLCV in tomato plants

The number of DNA copies of TYLCV and derivatives was assessed with a standard curve obtained with a serial tenfold dilution of the plasmid p1.01TYLC (8.45 × 10^3^–8.45 × 10^8 ^copies) in a 1/100 dilution of a mock-DNA extract from healthy tomato plants. The number of actin2 gene was assessed with a standard curve obtained with a serial tenfold dilution of a plasmid containing a PCR-amplified fragment (214 nt) of the actin2 gene (3.22 × 10^2^-3.22 × 10^7 ^copies). Results were expressed as the log of the ratio of the quantity of TYLCV to that of actin2.

### Statistics

Dry weight and virus accumulation at 15, 25 and 35 days after inoculation were compared using an ANOVA with the R statistical software (R Development Core Team).

## Competing interests

The authors declare that they have no competing interests.

## Authors' contributions

CU carried out the virus extraction, cloning of progeny, sequence analysis, phenotypic experimentations and drafted the manuscript. GT performed the statistical analysis. MG carried out the plasmid and viral construction. MP designed the molecular construction. SB and MP conceived of, designed and coordinated the study, and helped to draft the manuscript. All authors read and approved the final manuscript.
